# Employment status transitions in employees with and without chronic disease in the Netherlands

**DOI:** 10.1007/s00038-018-1120-8

**Published:** 2018-05-30

**Authors:** Angela G. E. M. de Boer, Goedele A. Geuskens, Ute Bültmann, Cécile R. L. Boot, Haije Wind, Lando L. J. Koppes, Monique H. W. Frings-Dresen

**Affiliations:** 1 Coronel Institute of Occupational Health, Academic Medical Center, Amsterdam Public Health Research Institute, PO Box 22700, 1100 DE Amsterdam, The Netherlands; 2 Division Work and Employment, TNO, Hoofddorp, The Netherlands; 3 Department of Health Sciences, Community and Occupational Medicine, University Medical Center Groningen, University of Groningen, Groningen, The Netherlands; 4 Department of Public and Occupational Health, EMGO Institute for Health and Care Research, VU University Medical Center, Amsterdam, The Netherlands; 5 NIVEL, Netherlands Institute for Health Services Research, Utrecht, The Netherlands

**Keywords:** Employment, Unemployment, Work, Chronic disease, Self-employment

## Abstract

**Objectives:**

Objectives were to: (1) longitudinally assess transitions in employment status of employees with and without chronic disease; and (2) assess predictors of exit from paid employment.

**Methods:**

Transitions in employment status at 1- and 2-year follow-up were assessed in a longitudinal cohort study of employees aged 15–63 years. Generalised estimating equations (GEE) and logistic regression analyses were performed to analyse differences in transitions and identify sociodemographic, health- and work-related predictors.

**Results:**

At 1- and 2-year follow-up, 10,038 employees (37% with chronic disease) and 7636 employees responded. Employees with chronic disease had higher probability of leaving paid employment [OR 1.4 (1.1–1.6)] and unemployment, disability pension and early retirement. Employees without chronic disease had higher chance of moving into self-employment or study. At 2-year follow-up, employees with cardiovascular disease (15%), chronic mental disease (11%), diabetes (10%) and musculoskeletal disease (10%), had left paid employment most often. Higher age, poor health, burnout, low co-worker support and chronic disease limitations were predictors for leaving paid employment.

**Conclusions:**

Employees with chronic disease leave paid work more often for unfavourable work outcomes.

## Introduction

In recent years, improvements in the treatment and prognosis of many chronic diseases have resulted in increasing numbers of people living with a chronic disease (Huygens et al. 2016). About 28% of Europeans report having a chronic health problem or illness (Corral et al. 2014), while in the USA, half of the population of working-age adults have chronic health care needs (Gulley et al. 2011). The prevalence of working-age people with a chronic disease is expected to grow further in most industrialized countries because of an ageing population, continued improvements in treatment and recent legislations in many countries to raise the retirement age (Boot et al. 2014).

The majority of employees with a chronic disease experience physical, emotional and social problems such as fatigue, pain, depression, diminished physical or cognitive functioning, which may result in limitations in social activities (Varekamp et al. 2011) including work. Perceived poor health is strongly associated with unemployment in most European countries (Ranzi et al. 2013), and people with chronic diseases such as stroke, myocardial infarction, angina (Nakaya et al. 2016) and cancer (Nakaya et al. 2016; de Boer et al. 2009) are significantly less often in paid employment. However, these studies were all cross-sectional and did not include transitions in employment status.

Results from earlier studies (Schuring et al. 2013; van den Berg 2010; Reeuwijk et al. 2017; Carr et al. 2018) have shown that having a chronic disease or poor health is related to exit from paid work. However, it is not yet clear if exit routes from paid employment are similar for people with and without a chronic disease. Exit from paid employment into unemployment, disability pension and early retirement may be one of the most stressful life events and can lead to diminished social status, disturbed social role patterns, financial hardship, reduced self-esteem, isolation and feelings of guilt (Audhoe et al. 2010; Hoving et al. 2013). Exit from paid employment into education or self-employment, on the other hand, may ultimately lead to an improved, more favourable work situation (Carr et al. 2018). In this study we will therefore study the differences in exit from paid employment trajectories for people with and without a chronic disease.

More knowledge on patterns and predictors of exit from paid employment of employees with and without a chronic disease is necessary to develop effective policies and interventions to sustain employment and prevent exit from paid employment. Low socio-economic status (Schuring et al. 2013), low education and low job control (van den Berg et al. 2010; Sewdas et al. 2018) are associated with early exit from paid employment in people with poor health; however, little is known about other sociodemographic, disease-related and work-related predictors of exit from paid employment. Furthermore, employees with and without a chronic disease might differ with regard to factors predicting exit from paid work because they might value aspects of work differently (de Jong et al. 2015).

The aims of this study were therefore: (1) to longitudinally assess transitions in employment status of employees with and without a chronic disease in the Netherlands; and (2) to assess predictors of exit from paid employment in employees with and without a chronic disease in the Netherlands.

## Methods

### Participants

Data from the Netherlands Working Conditions Cohort Study (NWCCS) were used (Koppes et al. 2010). The study was exempt from Medical Ethical Review.

### Design

We used longitudinal cohort data from NWCCS 2007 (baseline), 2008 and 2009. In 2007, 80,000 individuals were sampled from the Dutch working population database of Statistics Netherlands (van den Bossche et al. 2008). Sampling was random, except for a 50% over-sampling of employees aged younger than 25 years and with non-Western ethnic origin. Sampled individuals received the written questionnaire by postal mail at their home address (van den Bossche et al. 2008). In total, 33% of the employed sampled individuals responded to the baseline questionnaire (*n* = 21,747). Non-response analysis (Koppes et al. 2010) showed that more women than men responded, more people in the 45–64 age categories and more people with a higher education responded.

At baseline, all respondents were in paid employment. Paid employment included having an employer, a contract and a salary. The data collection after 1- and 2-year follow-up in 2008 and 2009 was similar to the data collection at baseline. Only individuals with an age of 62 years or younger in 2007 were selected in order to exclude those who would go into old age pension in 2008 or 2009 at the age of 65.

### Measurements

Work situation included having a paid employment (yes, no), unemployment, disability pension, early retirement pension, old age pension, study, self-employment, and other benefit or income, and number of working hours per week.

Sociodemographic factors included age (years), gender (male/female) and educational level (low, intermediate, high).

Chronic disease was defined as a disease which lasts more than 3 months. Chronic disease was measured with 12 questions (yes/no) and included: no chronic disease; chronic musculoskeletal disease (rheumatoid arthritis, arthrosis) of the arms or legs, migraine or severe headache; cardiovascular disease; asthma, bronchitis, or emphysema; chronic stomach or bowel disease; diabetes; severe chronic skin disorders; mental disease; epilepsy; other chronic disease (standardised questionnaire from the National Bureau of Statistics NL, 2004). Workers with one or more chronic disease were scored as ‘with chronic disease’.

Subjective health was measured with 1 item of the SF-36 on a 5-point scale (poor health—excellent health) (standardised question from the National Bureau of Statistics NL, 2004; Aaronson et al. 1998). Burnout complaints (feeling emotionally drained and physically exhausted) was measured with five items on a seven-point scale of the valid and reliable Maslach Burnout Inventory (MBI) (never always) (Schaufeli et al. 2006).

Work-related factors included shift work (often, sometimes, never) and work adjustments (yes, no). Perceived autonomy of the valid and reliable Job Content Questionnaire (JCQ) (Karasek et al. 1998) was measured with five items and time pressure with two items (standardised questionnaire from the National Bureau of Statistics NL 2004), all measured on a three-point scale (none, sometimes, often). Task demands (heavy workload and fast working) (standardised questionnaire from the National Bureau of Statistics NL 2004) were measured with four items, emotional job demands was measured with three items from the valid and reliable Copenhagen Psycho Social Questionnaire (COPSOQ) (Kristensen and Borg 2000), supervisor and co-worker social support were measured with the JCQ with four items each (Karasek et al. 1998), and these measures were scored on a four-point scale (never always). The mean value of the items in each measure is reported.

Respondents were asked if they expected to work until the age of 65 years (yes, don’t know, no). Respondents with a chronic disease were asked if their disease limits their work (no, somewhat, strongly). All questionnaires are reliable and valid and have been tested in international studies.

### Analysis

Differences in baseline data between employees with and without a chronic disease regarding sociodemographic, disease-related and work-related factors were analysed with Chi-square tests and *t* test for categorical and continuous factors, respectively. Differences in baseline data on age, gender and chronic disease between responders and non-responders at 1- and 2-year follow-up measurements were analysed with a *t* test and Chi-square tests.

To compare transitions in work status (leaving paid employment) of employees with and without a chronic disease, Chi-square tests were used. Second, we used generalised estimating equations (GEE) analyses using repeated measurements with a binary logistic model to model differences in changes of employment between employees with and without a chronic disease over 2 years. To analyse different developments between employees with and without chronic disease over time, the interaction between time*chronic disease was added to the model. Odds ratios (OR) with 95% confidence intervals (CI) were be reported.

A substantial reduction in working hours was calculated as ≥ 10% reduction in working hours and differences between employees with and without a chronic disease were analysed with Chi-square tests.

In the analyses of prognostic factors, exit from paid employment was defined as a transition into unemployment, disability pension or early retirement pension. Univariate logistic regression analyses were performed between exit from paid employment (yes, no) measured at 1- and 2-year follow-up and the baseline prognostic factors age, gender, educational level, number of working hours, shift work, work adjustments, perceived autonomy, time pressure, task demands, emotional demands, supervisor and co-worker social support, burnout complaints and ability to work until the age of 65 years. Separate analyses for the groups of employees with and without a chronic disease were executed.

Next, we entered all prognostic factors for which the estimates of the univariate logistic regression analyses had a *p* value of ≤ 0.10, into multivariate logistic regression analyses. The factors associated with exit from paid employment were selected for final multivariable model if they had a *p* value of ≤ 0.05 in the final model. Goodness of fit of the final multivariable models was tested with Hosmer and Lemeshow tests. Results were for 1-year follow-up *p* = 0.25 and *p* = 0.001 for people with and without a chronic disease, respectively, and for 2-year follow-up *p* = 0.12 and *p* = 0.013 for people with and without a chronic disease, respectively.

All analyses were performed for both follow-up measurements to analyse both short-term and long-term effects. Alpha was set at 0.05, and analyses were conducted with IBM SPSS 20.

## Results

At baseline 21,747 employees aged 15–62 years with paid work were included, while at 1- and 2-year follow-up, 10,038 (46%) and 7636 (35%) employees responded. Respondents were older (43 years) than non-responders (40 years) and more often had a chronic disease at baseline (37 vs 35%), but no gender differences (51% female) were found.

Table 1 shows sociodemographic, disease-related and work-related baseline characteristics of 3747 (37%) employees with a chronic disease and 6291 (63%) without a chronic disease who completed at least one follow-up questionnaire. Employees with a chronic disease were significantly older (45 vs 41 years), more often female (54 vs 51%) and significantly working less hours per week (31 vs 32 h). Significantly more employees with a chronic disease (21%) than those without (10%) had work adjustments. Employees with a chronic disease significantly reported lower scores on subjective health (2.1) than those without chronic disease (2.7, *p* < 0.001). Furthermore, 51% of those with a chronic disease indicated that their disease limited their work somewhat (44%) or strongly (7%).

**Table 1:** Baseline characteristics of *n* = 10,038 employees (Netherlands 2007)

	Chronic disease *n* = 3747	No chronic disease *n* = 6291
Age (range 15–62, mean, SD)	45.0 (10.9)	41.2 (11.3)***
Gender, female *n* (%)	2003 (54%)	3181 (51%)**
Education, *n* (%)		
Low	818 (22%)	1034 (17%)***
Middle	1484 (40%)	2466 (39%)
High	1427 (38%)	2759 (44%)
Number of working hours per week (mean, SD)	31 (9.6)	32 (9.6)*
Shift work, *n* (%)		
Yes, often	417 (12%)	709 (12%)
Yes, sometimes	64 (2%)	115 (2%)
Never	3145 (87%)	5285 (87%)
Autonomy in job (range 1–3, mean, SD)^b^	2.5 (0.5)	2.6 (0.5)***
Time pressure in job (range 1–3, mean, SD)^b^	2.2 (0.7)	2.2 (0.6)
Task demands (range 1–4, mean, SD)^b^	2.4 (0.6)	2.3 (0.6)*
Emotionally demanding job (range 1–4, mean, SD)^b^	1.9 (0.6)	1.8 (0.6)***
Social support manager (range 1–4, mean, SD)^b^	2.8 (0.7)	2.9 (0.6)***
Social support colleagues (range 1–4, mean, SD)^b^	3.2 (0.5)	3.3 (0.5)***
Burnout complaints (range 1–7, mean, SD)^b^	2.2 (1.3)	1.8 (0.9)***
Work adjustments (yes) *n* (%)	798 (21%)	617 (10%)***
Working possible until age 65 *n* (%)		
Yes	1449 (39%)	2934 (47%)***
Don’t know	793 (21%)	1239 (20%)
No	1480 (40%)	2094 (33%)
Subjective health (range 1–5, mean, SD)^a^	2.1 (0.8)	2.7 (0.8)***
Chronic disease limits work *n* (%)		–
No	1828 (49%)	
Somewhat	1628 (44%)	
Strongly	257 (7%)	

*SD* standard deviation

*p < 0.05; ** *p* < 0.01; ***p < 0.001

^a^Higher score indicates better health

^b^Higher score indicates more or higher levels

### Work status transitions and exit from paid employment

At 1-year follow-up, 4% of the employees with a chronic disease had left paid employment compared to 3% of the employees without a chronic disease, as depicted in Table 2. After 2 years, 8% of the employees with a chronic disease had left paid employment versus 6% of employees without a chronic disease (*p* < 0.01). Results from the GEE analyses with a binary logistic model which included those 10,038 employees with at least baseline and 1-year follow-up data showed that employees with a chronic disease had a higher chance of losing their job compared to those without a chronic disease (OR 1.4; 95% CI 1.1–1.6) but no interaction effects with time were found.

**Table 2:** Employment status transitions at 1- and 2-year follow-up in employees with and without a chronic disease (Netherlands 2008–2009)

*N* (%)	1-Year follow- up		2-Year follow-up		
	Chronic disease	No chronic disease	Chronic disease	No chronic disease	OR (95% CI)^a^
	*n* = 3747	*n* = 6291	*n* = 2875	*n* = 4761	*n* = 10,038
Paid employment	3600 (96)	6094 (97)	2659 (92)	4496 (94)	1.4 (1.1–1.6)
Exit from paid employment	147 (4)	197 (3)*	216 (8)	265 (6)**	
Reason of exit^b^	*n* = 147	*n* = 197	*n* = 216	*n* = 265	
Unemployment benefit	31 (21)	32 (17)	54 (25)	62 (23)	1.5 (1.0–2.2)
Disability pension	28 (19)	0 (0) ***	32 (15)	2 (1)***	38 (0.0– > 10^5^)
Early retirement pension	36 (25)	44 (23)	79 (37)	81 (31)	1.6 (1.2–2.2)
Old age pension	7 (5)	9 (5)	11 (5)	5 (2)*	3.6 (1.3–10.5)
Study	15 (10)	32 (17)	4 (2)	26 (10)***	0.3 (0.1–0.7)
Self-employed	14 (10)	52 (27)***	16 (7)	53 (20)***	0.5 (0.3–0.9)
Other benefit or income	25 (17)	36 (19)	35 (16)	52 (20)**	0.9 (0.7–1.1)

Chronic disease measured at baseline

*OR* odds ratio; *CI* confidence interval

*p < 0.05; **p < 0.01; ***p < 0.001 (Chi-square test)

^a^Results of GEE longitudinal measurements analyses

^b^More than one reason possible

The reasons for leaving paid employment were significantly different for those with and without a chronic disease. Significantly more employees with a chronic disease left because of receiving a disability pension compared to those without a chronic disease at both 1- and 2-year follow-up (19 vs 0% and 15 vs 1%, both *p* < 0.001). At both follow-up measurements, significantly fewer employees with a chronic disease had left paid employment because they had become self-employed (10 and 7%) compared to those without a chronic disease (27 and 20%, both *p* < 0.001). Results from the GEE analyses with a binary logistic model showed that employees with a chronic disease had a higher probability of unemployment (OR 1.5; 95% CI 1.0–1.6), disability pension (OR 38; 95% CI 1.0 to > 10^5^), early retirement (OR 1.6; 95% CI 1.2–2.2) and old age pension (OR 3.6; 95% CI 1.3–10.5), but a lower chance of becoming self-employed (OR 0.5; 95% CI 0.3–0.9) or going into study (OR 0.3; 95% CI 0.1–0.7).

Exit from paid employment at 1- and 2-year follow-up in employees < 63 years by chronic disease compared to no chronic disease is depicted in Table 3. Employees with epilepsy (11%), chronic mental disease (10%), cardiovascular disease (8%), severe skin disease (8%) and chronic musculoskeletal disease (rheumatoid arthritis, arthrosis) of the legs (6%) had left paid employment significantly (*p* < 0.05 to *p* < 0.001) more often than employees without a chronic disease (3%) at 1-year follow-up. At 2-year follow-up, significantly (*p* < 0.05 to *p* < 0.001) more employees with cardiovascular disease (15%), chronic mental disease (11%), diabetes (10%) and chronic musculoskeletal disease (rheumatoid arthritis, arthrosis) of the legs (10%), had left paid employment compared to employees without a chronic disease (6%).

**Table 3:** Exit from paid employment at 1- and 2-year follow-up in employees < 63 years by chronic disease compared to no chronic disease (Netherlands 2008–2009)

	1-Year follow-up *N* (%)		2-Year follow-up *N* (%)	
Chronic disease^a^	Paid employment	Exit from paid employment^b^	Paid employment	Exit from paid employment^b^
No chronic disease	6094 (97)	197 (3)	4497 (94)	264 (6)
Musculoskeletal disease (rheumatoid arthritis, arthrosis), arms	598 (96)	22 (4)	441 (94)	29 (6)
Musculoskeletal disease (rheumatoid arthritis, arthrosis), legs	459 (94)	28 (6)**	335 (90)	38 (10)**
Migraine	528 (96)	25 (4)	394 (93)	28 (7)
Cardiovascular disease	273 (92)	23 (8)***	200 (85)	36 (15)***
Asthma or COPD	518 (96)	20 (4)	375 (94)	25 (6)
Gastrointestinal disease	318 (95)	16 (5)	236 (91)	23 (9)
Diabetes	203 (95)	10 (5)	146 (90)	17 (10)*
Severe skin disease	79 (92)	7 (8)*	55 (93)	4 (7)
Chronic mental disease	225 (90)	24 (10)***	164 (89)	20 (11)**
Epilepsy	32 (89)	4 (11)*	27 (90)	3 (10)

*p < 0.05; ** *p* < 0.01; ***p < 0.001

^a^Measured at baseline in 2007

^b^Compared to job loss in employees without a chronic disease

A reduction in working hours of ≥ 10% at follow-up was more often reported by employees with chronic musculoskeletal disease (rheumatoid arthritis, arthrosis) of the legs (13%), chronic mental disease (13%) or asthma (12%) compared to those without a chronic disease (9%).

### Predictors for exit from paid employment

Result of univariate logistic regression analyses showed that for employees with a chronic disease, higher age, lower levels of social support from supervisor and co-workers, higher burnout score, lower subjective health score, a chronic disease strongly limiting work and a higher sickness leave percentage at baseline were predictive (*p* < 0.001) of exit from paid employment 1 year later (Table 4). Higher age, working under time pressure and not being able to work until 65 years of age were predictive for exit from paid employment for employees without a chronic disease at 1-year follow-up. At 2-year follow-up, higher age, being male, having more burnout complaints, lower subjective health and a chronic disease strongly limiting work were predictive of exit from paid employment for employees with a chronic disease and higher age for employees without a chronic disease.

**Table 4:** Univariate logistic regression associations between baseline characteristics and exit from paid employment due to unemployment, disability pension and early pension of employees with and without a chronic disease at 1- and 2-year follow-up (Netherlands 2008–2009)

	Exit from paid employment due to unemployment, disability pension and early pension, 1-year follow- up		Exit from paid employment due to unemployment, disability pension and early pension, 2-year follow-up	
Predictor at baseline	Chronic disease	No chronic disease	Chronic disease	No chronic disease
	*n* = 94/3747 OR (95% CI)	*n* = 76/6297 OR (95% CI)	*n* = 2875 OR (95% CI)	*n* = 4761 OR (95% CI)
Age	1.1*** (1.1–1.1)	1.2*** (1.1–1.2)	1.1*** (1.1–1.1)	1.2*** (1.1–1.2)
Gender (female)	0.7* (0.4–0.9)	0.8 (0.5–1.3)	0.5*** (0.4–0.7)	0.6* (0.4–0.8)
Education				
Low	1	1	1	1
Middle	1.0 (0.6–1.7)	0.7 (0.4–1.3)	0.6** (0.4–0.8)	0.6* (0.4–0.9)
High	0.8 (0.4–1.3)	0.6 (0.3–1.0)	0.5** (0.4–0.8)	0.5** (0.3–0.7)
Shift work (yes/no)	1.0 (0.7–1.4)	1.1 (0.8–1.6)	1.0 (0.8–1.3)	1.2 (0.9–1.6)
Autonomy in job (1–3)^b^	0.6** (0.4–0.8)	0.8 (0.5–1.4)	0.9 (0.6–1.2)	1.1 (0.8–1.6)
Time pressure in job (1–3)^b^	0.8 (0.6–1.1)	0.6** (0.4–0.9)	0.8* (0.6–0.98)	0.8* (0.6–0.98)
Task demands (1–4)^b^	1.0 (0.7–1.4)	0.7 (0.5–1.1)	0.9 (0.7–1.2)	0.8 (0.6–1.0)
Emotionally demanding job (1–4)^b^	1.2 (0.9–1.7)	1.0 (0.7–1.5)	1.0 (0.7–1.3)	0.7* (0.5–0.9)
Social support manager (1–4)^b^	0.6*** (0.4–0.8)	0.8 (0.6–1.1)	0.7** (0.6–0.9)	0.8 (0.6–1.0)
Social support colleagues (1–4)^b^	0.4*** (0.3–0.6)	0.7 (0.4–1.1)	0.7* (0.5–0.9)	0.6** (0.4–0.9)
Burnout complaints(1–7)^b^	1.5*** (1.3–1.6)	1.1 (0.9–1.3)	1.3*** (1.1–1.4)	0.9 (0.8–1.1)
Work adjustments (yes)	1.6* (1.0–2.5)	0.4 (0.1–1.2)	1.1 (0.8–1.6)	0.6 (0.3–1.2)
Ability work until age 65				
Yes	1	1	1	1
Don’t know	1.6 (0.8–3.0)	0.3** (0.1–0.7)	0.9 (0.6–1.5)	0.5* (0.3–0.9)
No	2.8** (1.7–4.7)	1.3 (0.8–2.0)	1.5* (1.0–2.1)	1.1 (0.7–1.5)
Subjective health (1–5)^a^	0.5*** (0.4–0.7)	1.0 (0.7–1.3)	0.6*** (0.5–0.7)	1.0 (0.8–1.2)
Chronic disease limits Work				
No	1	NA	1	NA
Somewhat	1.0 (0.6–1.6)		0.8 (0.6–1.2)	
Strongly	5.6*** (3.3–9.4)		3.2*** (2.0–5.1)	
Sick leave percentage	1.02*** (1.01–1.03)	0.99 (0.9–1.0)	1.01** (1.0–1.02)	0.98 (0.9–1.0)

Job loss includes unemployment, early retirement and work disability pension; all predictors measured at baseline

*NA* not applicable

*p < 0.05; **p < 0.01; ***p < 0.001

^a^Higher score indicates better health

^b^Higher score indicates more often or higher levels

Multivariate models for predictors of exit from paid employment are presented in Table 5. For employees with a chronic disease, higher age, higher sickness leave percentage at baseline and the extent to which the chronic disease limits work were predictors of exit from paid employment at both 1- and 2-year follow-up measurements. Lower levels of supervisor and co-workers social support and a higher burnout score predicted exit from paid employment at 1-year follow-up. Poor subjective health at baseline was predictive at 2-year follow-up. For employees without a chronic disease, higher age and the expectation of not being able to work until the age of 65 years predicted exit from paid employment at follow-up. Additionally, time pressure predicted exit from paid work at 1-year follow-up and emotional job demands at 2-year follow-up.

**Table 5:** Multivariate logistic regression models of predictors for exit from paid employment due to unemployment, disability pension and early pension, of initially employed people with and without a chronic disease at 1- and 2-year follow-up (Netherlands 2008–2009)

	Exit due to unemployment, disability pension and early pension, 1-year follow- up		Exit due to unemployment, disability pension and early pension, 2-year follow-up	
Predictor at baseline	Chronic disease	No chronic disease	Chronic disease	No chronic disease
	*n* = 3747	*n* = 6297	*n* = 2875	*n* = 4761
	OR (95% CI)	OR (95% CI)	OR (95% CI)	OR (95% CI)
Age	1.1* (1.1–1.1)	1.1** (1.1–1.2)	1.1* (1.1–1.1)	1.2** (1.1–1.2)
Support	0.5*** (0.3–0.8)			
Colleagues				
Burnout complaints	1.2* (1.0–1.4)			
Sick leave %	1.02* (1.0–1.0)		1.01* (1.0–1.0)	
Subjective health			1.5** (1.1–1.9)	
Disease limits work				
No	1		1	
Somewhat	0.6* (0.4–1.0)		0.6* (0.4–0.96)	
Strongly	1.6 (0.8–3.5)		1.3 (0.7–2.6)	
Time pressure		0.5*** (0.4–0.8)		
Emotionally Demanding job				0.6** (0.5–0.9)
Ability work until age 65				
Yes		1		1
Don’t know		0.4 (0.1–1.1)		0.6 (0.3–1.1)
No		2.2*** (1.3–3.7)		1.7*** (1.1–2.5)

*p < 0.05; **p < 0.01; ***p < 0.001

## Discussion

We found that employees with a chronic disease had a higher probability of leaving paid employment compared employees without a chronic disease due to unemployment, early retirement or receiving a (disability) pension in the Netherlands. Moreover, employees *without* a chronic disease had left paid employment more often because they became self-employed or went into study. Employees with a chronic disease who have low co-worker support, more burnout complaints, report poor subjective health and have a chronic disease limiting work were at probability of leaving paid employment due to unemployment, disability pension and early retirement pension. In contrast, employees without chronic disease are at probability of leaving paid employment when they indicated not being able to work until the age of 65, having a high time pressure and having an emotionally demanding job. Higher age was a predictor for both groups.

### Strong points and limitations

A major strength of our study is the large number of employees with and without a chronic disease sampled from the Dutch national labour force registration. Furthermore, the longitudinal design of the study enabled us to examine the patterns of employment transitions over time and to determine predictors measured at baseline on subsequent exit from paid employment due to unemployment, disability pension and early pension at follow-up. Therefore, the causal relationship between poor health and unemployment has been more strongly indicated in this study compared to earlier, cross-sectional, studies. Furthermore, predictors of exit from paid employment could already be assessed both one and two years earlier.

The limitations of our study are, firstly, the high attrition rates between baseline and follow-up measurements of 54 and 65%. This might be due to the recruitment strategy in which respondents were asked at baseline to indicate if they agreed to be contacted again. The baseline cohort therefore included participants who were not willing to complete follow-up questionnaires. Second, all analyses of this study were based on self-reported data including chronic disease. Although self-reported health is regarded as a valid measure (Wannamethee and Shaper 1991), a possible bias (common method variance) cannot be excluded. Hence, the number of people with a chronic disease might be overrepresented in this study. We do, however, think that this potential overestimation will not have affected the estimation of trajectories or predictors in this study.

### Interpretations

During the 2-year follow-up period, 6% of all employees had left paid employment. This percentage is lower than in a previous SHARE study performed in 11 European countries (van den Berg et al. 2010) which found that 17% of employees had left paid employment in 2 years. However, the European SHARE study was focused on workers of 50 years or older and therefore higher exit rates are to be expected.

The transitions of leaving paid employment in our study were different for employees with and without a chronic disease. The finding that more employees with a chronic disease received a disability pension at follow-up is in line with earlier studies (Nakaya et al. 2016; Alexanderson et al. 2012). In addition, more employees with a chronic disease left paid employment because of early retirement or unemployment which confirms earlier Dutch and Swedish studies on people with chronic diseases (van den Berg et al. 2010; Lindbohm et al. 2001).

It was an innovative finding that leaving paid employment to become self-employed was more prevalent in employees without a chronic disease. Becoming self-employed involves renouncing the certainty of a regular income and the benefits of paid sick leave and this could explain why employees with a chronic disease are less likely to choose self-employment. Earlier research found that self-employment seems to protect against disability pension (Ropponen et al. 2014).

An important predictor of exit from paid work in employees with a chronic disease was co-worker social support. The importance of having more psychosocial resources and support of co-workers and supervisors on having paid work and less sick leave is confirmed in Dutch and Hong Kong studies (Boot et al. 2014; Flach et al. 2013; Siu et al. 2013). The support of colleagues therefore seems to create a buffer between the adverse effects of a chronic disease at the workplace and negative work consequences such as unemployment and disability pension.

### Implications for research and practice

Policies and interventions should be developed to enable employees to remain in paid employment. These interventions should especially aim at employees with a chronic disease with a high chance of unemployment, early retirement, or disability pension: older employees, employees with high sick leave, more burnout complaints, or poor subjective health. Furthermore, policies and interventions should target those who indicate that their chronic disease limits work or that they receive little support from colleagues. Rehabilitation specialists, occupational physicians, supervisors and personnel advisers could play an important role in detecting those employees at risk, because our study has shown that the indication of employees with a chronic disease with future probability of exit from paid work can be assessed years before actual exit from paid employment occurs. A possible intervention could be the provision of work adjustments for employees with a chronic disease because our earlier study showed that work adjustment for employees with a chronic disease significantly reduced sick leave (Boot et al. 2013). Another possibility to reduce the probability of early exit from paid work would be to aim at the colleagues of employees with a chronic disease in helping them to support their colleague with a chronic disease.

Remarkably, employees with a chronic disease were less likely to become self-employed compared to those without a chronic disease. It is possible that they would have preferred to start self-employment as well but did not have the opportunity, capability or resources. In that case, policies to support them to start their own company or become self-employed may provide a support to employees with a chronic disease. Future research should involve a longer follow-up period regarding exit from paid employment with more measurements of predictive factors to examine (cumulative) effects over time because a relatively small percentage of all employees leaves paid employment each year.

## Conclusion

Employees with a chronic disease have a higher probability of leaving paid employment compared employees without a chronic disease. More employees with a chronic disease left paid employment because they were unemployed, on early retirement or receiving a (disability) pension. In contrast, employees without a chronic disease left paid employment more often because they became self-employed or went into study. New interventions and policies to prevent leaving paid employment should especially aim at: older employees, employees with high sick leave, more burnout complaints, poor subjective health and employees who indicate that their chronic disease limits work or that they receive little support from colleagues. If these supportive interventions and policies are effective, the sustainability of employment and quality of (working) life, financial situation and future work ability of employees with a chronic disease might be enhanced.
